# Community Dialogue to Shift Social Norms and Enable Family Planning: An Evaluation of the Family Planning Results Initiative in Kenya

**DOI:** 10.1371/journal.pone.0153907

**Published:** 2016-04-28

**Authors:** Christina Wegs, Andreea A. Creanga, Christine Galavotti, Emmanuel Wamalwa

**Affiliations:** 1 Sexual, Reproductive and Maternal Health Team, CARE USA, Atlanta, Georgia, United States of America; 2 Department of International Health, Johns Hopkins Bloomberg School of Public Health, Baltimore, Maryland, United States of America; 3 International Center for Maternal and Newborn Health, Johns Hopkins Bloomberg School of Public Health, Baltimore, Maryland, United States of America; 4 Health Sector Team, CARE Kenya, Nairobi, Kenya; University of Aberdeen, UNITED KINGDOM

## Abstract

**Introduction:**

Use of family planning (FP) is powerfully shaped by social and gender norms, including the perceived acceptability of FP and gender roles that limit women’s autonomy and restrict communication and decision-making between men and women. This study evaluated an intervention that catalyzed ongoing community dialogues about gender and FP in Siaya county, Nyanza Province, Kenya. Specifically, we explored the changes in perceived acceptability of FP, gender norms and use of FP.

**Methods:**

We used a mixed-method approach. Information on married men and women’s socio-demographic characteristics, pregnancy intentions, gender-related beliefs, FP knowledge, attitudes, and use were collected during county-representative, cross-sectional household surveys at baseline (2009; n_11_ = 650 women; n_12_ = 305 men) and endline (2012; n_21_ = 617 women; n_22_ = 317 men); exposure to the intervention was measured at endline. We assessed changes in FP use at endline vs. baseline, and fitted multivariate logistic regression models for FP use to examine its association with intervention exposure and explore other predictors of use at endline. In-depth, qualitative interviews with 10 couples at endline further explored enablers and barriers to FP use.

**Results:**

At baseline, 34.0% of women and 27.9% of men used a modern FP method compared to 51.2% and 52.2%, respectively, at endline (p<0.05). Exposure to FP dialogues was associated with 1.78 (95% CI: 1.20–2.63) times higher odds of using a modern FP method at endline for women, but this association was not significant for men. Women’s use of modern FP was significantly associated with higher spousal communication, control over own cash earnings, and FP self-efficacy. Men who reported high approval of FP were significantly more likely to use modern FP if reporting high approval of FP and more equitable gender beliefs. FP dialogues addressed persistent myths and misconceptions, normalized FP discussions, and increased its acceptability. Public examples of couples making joint FP decisions legitimized communication and decision-making with spouses about FP especially for men; women described partner support as key enabler of FP use.

**Conclusions:**

Our evaluation demonstrates that an intervention that catalyzes open dialogue about gender and FP can shift social norms, enable more equitable couple communication and decision-making and, ultimately, increase use of FP.

## Introduction

Globally, an estimated 225 million women have unmet need for family planning [1]. Increasing acceptance and use of family planning requires more than increasing access to health services: truly effective family planning programs must address the social and gender norms that present critical barriers to sexual and reproductive health. Use of family planning is powerfully shaped by social norms, including perceived acceptability of family planning, social pressure for large families, and perceived opposition to family planning by religious and community leaders and spouses [2].

Rigid gender roles and unequal power between men and women inhibit couple communication and joint decision-making about family planning: power dynamics in couples have been found to influence the use of family planning and other health services [3–5]. Fear of and experience with intimate partner violence or gender-based violence are barriers to contraceptive use [6–9]. Women who report intimate partner violence are at higher risk for unintended pregnancy than women who do not report violence [8]. Analyses of data from Demographic and Health Surveys (DHS) in a number of African countries suggest that key dimensions of gender equity and women’s empowerment—including equitable beliefs about gender roles, women’s ability to negotiate sexual activity, and women’s control over household economic decision-making—are associated with higher contraceptive use [10]. Several studies have also found a positive association between spousal communication and the use of contraception [11–14].

Despite evidence demonstrating the important relationships between gender, women’s empowerment, and family planning, there is little consensus about how to best measure these complex social constructs. Researchers have attempted to measure specific domains of women’s empowerment, and several scales have been developed including measures of mobility [15], power in relationships [3], gender norms and decision-making autonomy [16], control over assets [17], and social capital [18,19]. The lack of a standard set of widely used measures for women’s empowerment, however, hampers our ability to compare program effects across studies, populations, and cultural contexts.

The objective of the research described in this article was to evaluate the impact of a CARE intervention that catalyzed community-level dialogue about gender, sexuality, and family planning on household-level gender dynamics and reported use of family planning among men and women in Kenya. For this evaluation, we used several scales from WE-MEASR (Women’s Empowerment-Multidimensional Evaluation of Agency, Social Capital, and Relations) (see S1 File), a new tool that CARE developed following multi-year research into the effects and impact of our women’s empowerment programming [20].

## Methodology

### Study Setting

According to the 2008–09 Kenya DHS, approximately one in four women has an unmet need for family planning [21]. A 2013 analysis of DHS data found that Kenyan women who do not use family planning report method-related concerns, especially fear of harmful side effects, as the most common reason for non-use (43%); opposition to family planning (both the perceived religious prohibition of family planning and opposition by husbands/partners) is the second most commonly cited reason (16%) [22].

CARE conducted the research described here as part of the Family Planning Results Initiative (FPRI), which we implemented in Siaya County, Nyanza province, Kenya from July 2009 through December 2012. The majority of the county’s population of 842,304 is under 30 years old, and 46.1% are between 0 and 14 years of age [23]. Over 90% of Siaya County is rural and, like the rest of Nyanza, falls behind the national average on various indicators of development, gender inequality, poverty, education, and health, including infant and under-five mortality, antenatal care coverage, and HIV prevalence [21]. The contraceptive prevalence rate among married women in Nyanza province is among the lowest in Kenya: 37.3% of women use any method (compared to 45.5% nationally), and only 32.9% use a modern method (39.4% nationally) [21].

### The Intervention

CARE used its *Social Analysis and Action* approach [24] to shape FPRI’s central intervention about 150 community-based facilitators were trained and held ongoing community dialogues about gender, sexuality, and family planning over three and a half years (July 2009-December 2012). At any one time, an average of 65 facilitators were actively convening dialogues. Monthly planning meetings were held to plan activities and ensure adequate location coverage and topic variation over the entire study area. On a quarterly basis, CARE observed and provided feedback on dialogues and completed progress reviews with facilitators. Based on our monitoring data, CARE organized 759 dialogues between July 2009 and December 2012. Dialogues were held in a variety of venues and were promoted by community leaders or organized around pre-scheduled community meetings. Based on proximity to a respective venue, they were attended by participants from several villages, and, many times, were included in villages’ development plans.

The dialogues were designed to normalize communication about sensitive topics like gender norms and FP, and to catalyze participants’ critical analysis of how gender norms and dynamics restrict family planning acceptability and use. During dialogues, community leaders and satisfied family planning users acted as role models and overtly expressed support for family planning, equitable gender norms, open communication and shared decision-making regarding family planning between spouses.

Trained, community-based facilitators convened dialogues in an array of settings, such as markets, churches, women’s groups and village meetings. Local theatre groups often performed during the dialogues. Facilitators included community health care workers, religious leaders, local government officials, and teachers. CARE provided ongoing support to facilitators in the form of training and supervision, and also provided transport reimbursement and lunch/refreshments on days when dialogues or trainings were conducted; no incentives were provided for participation in intervention activities to any other community members.

All of CARE’s activities were coordinated with the District Health Medical Team (DHMT.) During the intervention period, the DHMT coordinated several interventions designed to increase availability and access to contraceptives, including in-service training for providers, strengthening of systems for ordering contraceptives, and some community outreach activities (FP counseling and method provision). To our knowledge, no other similar FP dialogue interventions or activities were implemented by other organizations during the intervention period.

### Theory of Change

**Fig 1:**
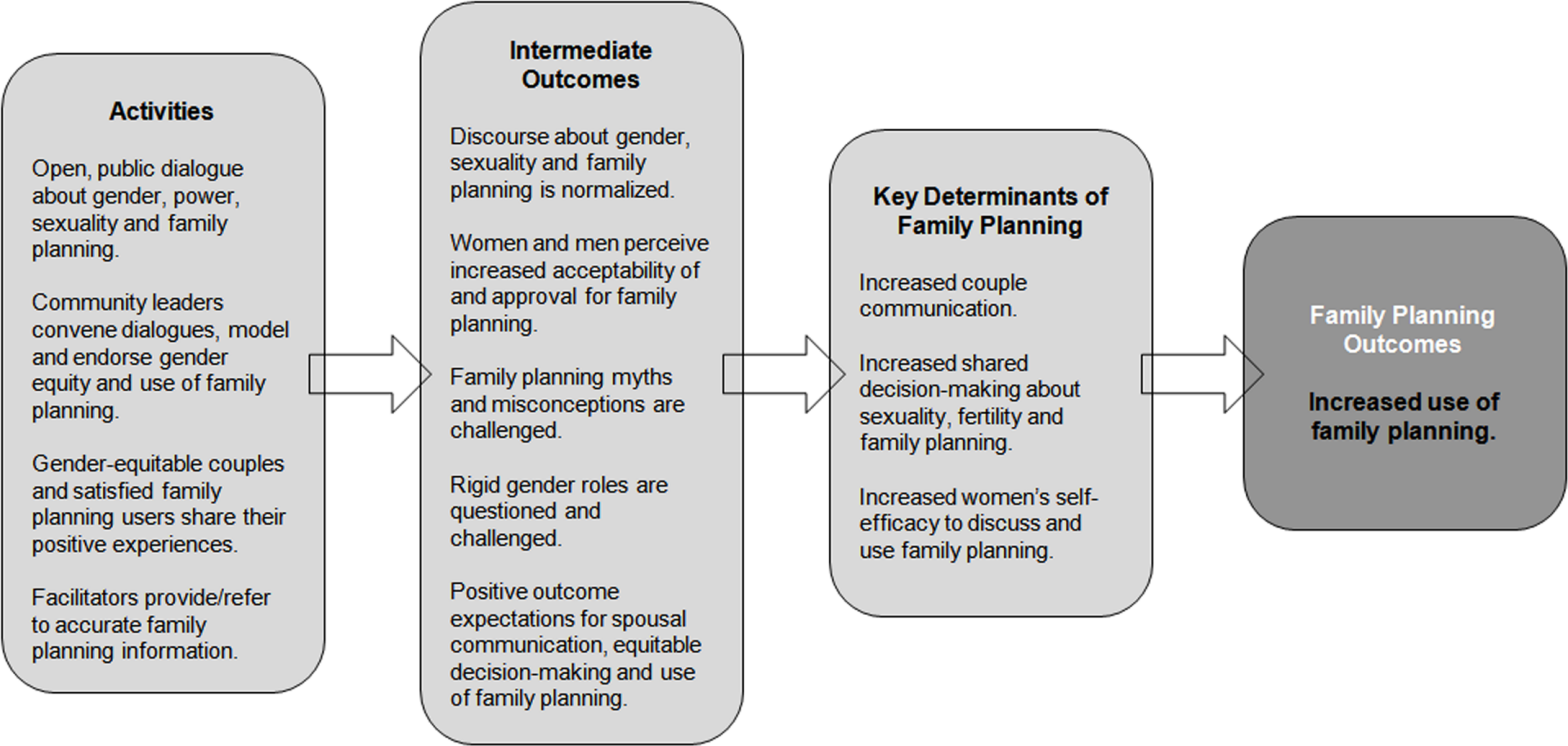
CARE’s Family Planning Results Initiative Theory of Change. This theory of change illustrates, from left to right, the activities undertaken as part of CARE’s Family Planning Results Initiative, the expected intermediate outcomes that will lead to improvements in three key determinants of family planning which, ultimately, increase use of family planning in the intervention community.

The intervention aimed to challenge and shift key community norms about gender dynamics and family planning, with the ultimate goal of creating a social environment that is more supportive of equitable gender relations and the use of family planning. Our research goals were to determine whether and how the ongoing dialogues shifted social norms, and whether and how these shifts at the community level influenced communication, decision-making, and family planning use at the couple or household level. Our theorized pathway of change is shown in Fig 1.

### Evaluation Design

We used both qualitative and quantitative methods to evaluate the intervention. At baseline (February 2009) and again at endline (December 2012), we conducted household surveys to collect data about men and women’s socio-demographic characteristics, pregnancy intentions, family planning knowledge, beliefs, attitudes, and use. At endline only, we measured key gender-related beliefs and behaviors and exposure to the intervention, which enabled us to determine whether these were important predictors of family planning use. Also at endline, we conducted in-depth, qualitative interviews with purposively selected couples from the intervention area (described below) to explore changes in communication, decision-making, and gender roles over the previous four years and to identify specific enablers and barriers to family planning use.

### Ethics Statement

CARE obtained approval to conduct this research from the Institutional Research and Ethics Committee at Moi University in Eldoret, Kenya.

### Quantitative Data Collection

Through independent, cross-sectional household surveys, married men (20–49 years) and women (18–45 years) were interviewed by trained interviewers using standardized questionnaires at baseline and endline. Although the two samples were entirely independent (no attempt was made at endline to include or exclude respondents who participated at baseline), both were drawn from the same Siaya county using an identical two-stage cluster sampling approach. Separately at baseline and endline, thirty sub-counties were randomly selected from Siaya’s 130 sub-counties, and 60 villages were randomly selected from this sub-set of sub-counties (2 villages from each sub-county,). Households were then randomly selected for interviews using a systematic approach, selecting every 3^rd^ household for women and every 5^th^ household for men. Respondents were eligible for interview if they were married or in union and of reproductive age, as defined above. If household member listing revealed more than one woman/man meeting the eligibility criteria, one interviewee was randomly selected in each household. A total of 1,240 women (n = 650 at baseline, n = 590 at endline) and 590 men (n = 305 baseline, n = 285 endline) completed interviews. Data were double-entered and cleaned by trained clerks under the supervision of a data manager.

### Qualitative Data Collection

To further explicate the quantitative findings, we used qualitative methods to explore gender dynamics and influences on family planning use at the couple level. Data were collected using face-to-face, in-depth interviews, and a visual timeline tool to elicit relationship histories and to explore changes in communication, decision-making, and gender roles during the previous four years.

FPRI project staff identified men and women from married couples in the intervention county, and conducted interviews until saturation on key themes was reached (n = 10 couples). In each couple, one or both partners were between the ages of 18 and 49 and had participated in a minimum of three intervention dialogues between 2009 and 2012. Interviews were conducted separately with the man and woman of each couple; each was interviewed by a trained, gender-matched interviewer in the Luo language. Interviews were recorded, detailed notes were taken by a trained observer during the interview, and recordings were used to transcribe, review, and confirm completeness and accuracy of notes. To help ensure data integrity, interviewers completed written summaries on the same day as data collection. Two senior researchers confirmed completeness and clarity of notes from each interview. Narratives from each of the men’s and women’s interviews were then analyzed and triangulated to create a narrative summary of each couple’s relationship and history of family planning use. After each couples’ story was analyzed, content analysis was conducted across all narrative summaries to identify key themes related to the theory of change.

### Quantitative Measures

#### Family Planning Measures (baseline and endline)

At both baseline and endline, men and women were asked identical questions about family planning knowledge, beliefs, attitudes, and use in order to measure changes in family planning over time (see S2 File).

#### Family Planning Knowledge, Beliefs and Attitudes (Independent Variables)

Men and women were asked to report the total number of family planning methods known, and if they knew where to obtain a method of family planning.

Beliefs about family planning were measured using a 4-item index, in which men and women reported whether they disagreed (scored as 1), were unsure (scored as 2), or agreed (scored as 3) with four common family planning-related myths. The index score range is 4–12, and a higher score indicates less accurate knowledge about family planning.

An index assessed men’s and women’s approval of family planning use in 5 different circumstances, with each response scored as 1 if the respondent approves and 0 if he/she does not approve. The index was constructed by summing the item scores. The index score range is 1‐5, and a higher score indicates a higher level of approval for family planning. Men and women were also asked if they believed it was acceptable for women to use a family planning method without their partner/husbands’ permission, and women (but not men) were asked if they believed they could suggest using a condom to their partner/husband.

#### Family Planning Use (Dependent Variable)

Men and women were asked if they or their spouse had ever used a method to avoid or delay pregnancy; those who answered ‘yes’ were asked to list all the methods they had ever used. Men and women were also asked if they or their spouse were currently using a method to avoid or delay pregnancy; those who answered ‘yes’ were asked to list all the methods they were currently using. These data were used to create two dependent variables: *current use of any contraceptive method* and *current use of a modern contraceptive method* (oral contraceptives, male/female condoms, vasectomy and tubal ligation, intrauterine device, injectable contraception, hormonal implants).

#### Exposure, Pregnancy Intentions and Gender Measures (Endline only)

In order to measure key predictors of family planning use, at endline we asked men and women questions about their exposure to the intervention as well as their pregnancy intentions. We also used several indices and scales from CARE’s WE-MEASR tool to measure key dimensions of gender equity and women’s empowerment in the endline survey: men’s belief in traditional gender roles (asked of men only), self-efficacy to use family planning (asked of women only), spousal communication and women’s decision-making power (asked of both sexes). The WE-MEASR scales used in the evaluation were tested and confirmed to be statistically reliable for use with both men and women in this sample.

#### Exposure to the intervention

We asked men and women about their exposure to the intervention between 2009 and 2012. To ensure that reported exposure did represent participation in the intervention, FPRI managers suggested unique indicators of exposure, including a mix of dialogue topics unique to the intervention. The three exposure variables were: exposure to any dialogues (yes/no), the number of topics exposed to during the dialogues (#), and exposure to dialogues specifically focused on family planning (yes/no).

#### Pregnancy intentions

At endline, we asked men and women whether they would like to have a child/another child, no more children, could not become pregnant, or were unsure. Those who reported wanting another child or being unsure were asked if they would like to get pregnant in the next 12 months, after 1–2 years, after more than 3 years or don’t know. Men and women who did not want to have children or were not able to have children (due to sterilization or infertility) were not asked this question.

Women/couples who were currently pregnant were also asked if they wanted to have another child after the child they were expecting now, wanted no more children or were unsure. Those who reported wanting another child or being unsure were asked how long they wanted to wait to have another child: within 12 months of the birth of the child they were expecting now, after 1–2 years, after more than 3 years, or don’t know.

#### Men’s beliefs about gender (men only)

We used a 9-item scale to measure how much men agreed or disagreed with statements about the roles and entitlements of men and women. We used a 5-point Likert scale, where Strongly Agree = 5, Agree = 4, Neither Agree Nor Disagree = 3, Disagree = 2, and Strongly Disagree = 1. The scale score was the sum of item scores divided by the number of items. The scale score range was 1‐5, and a higher scale score indicated a higher support for traditional gender roles (male dominance). Scale reliability in this sample was good (Chronbach’s alpha = .66).

#### Spousal communication (men and women)

We used a 5-item scale with both men and women to measure interspousal communication across a range of topics. Men and women were asked to report how often they communicated about: what had happened during their day, their worries and fears, their household finances, their fertility intentions/desired family size, and use of family planning. We used a 5‐point Likert scale, where Always = 5, Often = 4, Sometimes = 3, Seldom = 2 and Never = 1. The scale score was the sum of item scores divided by the number of items. The scale score range was 1‐5, and a higher scale score indicated a higher level of interspousal communication. Scale reliability for both samples was good (men: Chronbach’s alpha = .63; women: Chronbach’s alpha = .75).

#### Women’s decision-making power (men and women)

We measured women’s decision-making power in the household by asking both men and women who usually made a range of key household decisions. The scale was not intended to measure a woman’s decision‐making power over a specific kind of decision (e.g. household finances), but rather measure a woman’s influence over a range of key decisions that affect her life. We used slightly different scales for men and women, as detailed below.

We used a 12-item scale to ask women who usually made decisions about her health care, large household purchases, household purchases for daily needs, when she will visit family/relatives/friends, when the whole household will visit family/relatives/friends, how to use the money she brings into the household, how to use the money her spouse brings into the household, when to sell a large asset (e.g. cow), when to sell a small asset (e.g. chicken), whether she can work to earn money, when she and her husband have sex, and whether the woman and her husband use family planning. Item response options included: wife alone, wife and husband together, husband alone, mother‐ or father‐in‐law, someone else, and mother or father. Responses of wife alone or wife and husband together were scored as a 2 and all other responses were scored as a 1. The scale score was the sum of item scores divided by the number of items. The scale score range was 1‐2, and a higher scale score indicated women’s self-perceived higher decision-making power. Reliability of this scale for women was good (Chronbach’s alpha = .74).

With men, we used a similar, 10-item scale that included all items described above, except whether women can work to earn money and who makes decisions about women’s health care, The scale score range was 1‐2, and a higher scale score indicated men’s perceptions of women’s higher decision-making power. The reliability of this scale for men was also good (Chronbach’s alpha = .79).

#### Women’s self-efficacy to use family planning (women only)

We used a 4-item scale to measure women’s self-efficacy. Women were asked how much they agreed or disagreed with four statements about how sure they felt that they could: discuss family planning with their husband, tell their husband they wanted to use family planning, use family planning, and use family planning without their spouses’ approval. We assessed responses based on a 5‐point Likert scale, where Completely Sure = 5, Somewhat Sure = 4, Neither Sure/Unsure = 3, Somewhat Unsure = 2, and Not at all Sure = 1. The scale score was the sum of item scores divided by the number of items. The scale score range was 1‐5, and a higher scale score indicated higher self‐efficacy to discuss and use family planning. We found this to be a reliable scale (Chronbach’s alpha = .75).

### Analyzing Changes in Family Planning Use

Sample characteristics were compared between baseline and endline using chi-squared tests for proportions and t-tests for means. We found several significant differences in the characteristics of the samples drawn at baseline and endline, and therefore used propensity scores to establish comparable samples at the two time points. Propensity scoring is a method of matching that uses available information on the characteristics of the study population to establish, in our case, matched pairs of baseline and endline survey respondents. To derive the propensity score used for matching, logistic regression models with survey round (endline vs. baseline) as the primary outcome variable were estimated separately for men and women using the following covariates: respondent’s age, age difference between respondent and partner, number of living children, education, partner’s education, whether the respondent works for cash, whether the woman makes decisions regarding use of any cash earned, pregnancy intentions, the number of family planning methods known, the summed score on the index assessing family planning beliefs, and the summed score on index assessing approval of family planning use. Only in the estimation models for men, we included knowledge of where to obtain family planning and score on a scale assessing beliefs about gender roles; and, only for women, we included the score on a family planning self-efficacy scale and a variable to assess whether they believed they could suggest the use of condoms to their partners. The predicted probabilities of being interviewed at endline vs. baseline from the models served as propensity scores, and the scores were then used with Stata’s psmatch2 command to examine the average difference in family planning use (any family planning method and modern family planning methods) between baseline and endline. After assessing the use of different propensity score matching techniques, we chose kernel matching due to its efficiency. Only propensity score values included in the common support region (the area in which the distribution of propensity scores for being interviewed at endline overlapped with the distribution of propensity scores for being interviewed at baseline) were used in the analysis. This excluded respondents who were least likely to produce a reliable baseline-endline match based on the observed characteristics, specifically, 35 of 1,240 women and 12 of 590 men in our samples. Characteristics of the study population before and after propensity score matching were assessed to ensure that the data were fully balanced with respect to the covariates used to derive the propensity scores.

### Analyzing Predictors of Family Planning Use at Endline

To explore associations between exposure to the intervention and use of family planning at endline, we used the endline survey data and fitted separate logistic regression models for two key family planning outcomes: current use of any family planning method and current use of a modern family planning method. The key exposure covariates of interest used in three separate models were: exposure to the intervention (yes/no), the number of discussion topics exposed to during the intervention, and exposure to family planning discussions during the intervention (yes/no). Our multivariate models also explored the associations between current use of family planning (any and modern methods) and other key potential predictors of family planning use, including: childbearing intentions, family planning knowledge and beliefs, men’s belief in traditional gender roles, men’s and women’s reports of spousal communication, men’s and women’s reports about women’s household decision-making power, and women’s self-efficacy to use family planning.

The multivariate analysis controlled for several background factors, including age of respondent (less than 25 years, 25–29 years, 30–34 years, 35 years or older), years of school completed by respondent and by partner, religion (Protestant, Catholic, or other), and number of living children. We also included a variable measuring difference in spousal age (0–5 years, 5–9 years, 10–14 years, and more than 15 years’ difference) which we hypothesized might magnify decision-making power differences between spouses. Analyses were conducted using Stata version 12.0.

## Results

### Quantitative Results

#### Characteristics of Survey Sample

Table 1 summarizes characteristics of the survey samples. A total of 1,240 women aged 15–45 (n = 650 at baseline, n = 590 at endline) and 590 men aged 18–49 (n = 305 baseline, n = 285 endline) completed interviews. At baseline, 55.6% of women interviewed were under the age of 29, as were 63.9% of women interviewed at endline. By contrast, only 34.7% of men at baseline and 44.9% of men at endline were under the age of 29. Most respondents did not report a large age difference between them and their spouses: 71.1% of women at baseline and 64.9% of women at endline reported age differences of fewer than 10 years.

**Table 1:** Sample characteristics.

Characteristics	Women			Men		
	Baseline N = 650	Endline^a^ N = 590	p-value^b^	Baseline N = 305	Endline^a^ N = 285	p-value^b^
**Socio-demographic**						
Age (years; %)						
<25	34.5	34.8	<0.001	13.1	17.9	0.042
25–29	21.1	29.1		21.6	27.0	
30–34	17.9	19.0		21.3	21.8	
≥35	26.6	16.4		43.9	33.3	
Age difference between male and female partners (years; %)^c^						
<5	30.3	31.0	0.030	41.0	41.4	0.962
5–9	40.8	33.9		38.4	39.7	
10–14	18.9	21.0		14.4	13.3	
≥15	10.0	14.1		6.2	5.6	
Number of living children						
Mean (std dev)^d^	3.5 (2.1)	3.3 (1.9)	0.025	3.4 (2.2)	2.8 (2.0)	0.001
Religion (%)						
Protestant	52.3	48.8	0.107	51.5	49.5	0.750
Catholic	27.1	25.6		30.5	31.9	
Other	19.2	24.8		16.1	17.5	
None/missing	1.4	0.9		2.0	1.1	
Education (years)						
Mean (std dev)	7.0 (3.0)	8.2 (3.0)	<0.001	8.0 (3.2)	8.9 (3.4)	<0.001
Partner education (years)						
Mean (std dev)	8.4 (2.9)	9.1 (3.3)	<0.001	7.2 (2.9)	8.3 (2.8)	<0.001
Works outside home for cash (%)						
Yes	39.1	32.4	0.014	61.3	60.4	0.057
**Women’s empowerment / Gender beliefs /Relationship with partner/pregnancy intentions**						
Woman makes decisions about spending the cash she earns (%)						
No cash earned	60.9	67.6	0.084	64.6	61.8	<0.001
All cash	27.2	21.7		29.5	11.9	
Some cash	10.6	9.8		5.9	16.5	
Missing	1.2	0.9		0.0	9.8	
Men’s gender beliefs scale score^e^						
Mean (std dev)				2.0 (0.04)	2.0 (0.03)	0.820
Participation in household decision-making scale score^f^						
Mean (std dev)		1.6 (0.2)			1.6 (0.3)	
Inter-spousal communication scale^g^						
Mean (std dev)		2.9 (0.3)			3.4 (0.8)	
Pregnancy intentions (%)						
Wants pregnancy in next 12 months	30.1	35.1	0.001	60.0	64.2	0.026
Wants to delay pregnancy for ≥ 1 year	21.2	26.6		16.17	9.8	
Wants no more children	23.9	21.7		4.3	8.4	
Does not think about/not sure/not applicable	24.8	16.6		19.7	17.5	
**Family planning knowledge/attitudes/subjective norms/ self-efficacy**						
Family planning beliefs score^h^						
Mean (std dev)	9.1 (1.6)	8.4 (1.9)	<0.001	8.8 (0.1)	8.4 (0.1)	0.005
# methods known^i^						
Mean (std dev)	3.2 (1.3)	3.9 (1.5)	<0.001	3.4 (0.1)	3.4 (0.1)	0.784
Believe woman can use family planning w/o partner’s permission (%)						
Agree		66.3			11.6	
Believe woman can suggest use of condoms (%)						
Agree	19.4	15.4	0.067			
Disagree/unsure	80.6	84.6	0.067			
Score on family planning use approval questions^j^						
Mean (std dev)	11.1 (2.0)	11.9 (2.0)	<0.001	11.63 (0.12)	10.82 (0.10)	<0.001
Family planning self-efficacy scale score^k^						
Mean (std dev)		4.2 (1.0)				
Knows where to get family planning (%)						
Yes					45.4	
**Exposure to intervention**						
Exposure to intervention, all topics (%)						
Yes		64.2			66.3	
Number of topics exposed to during intervention						
Mean (std dev)		1.5 (1.5)			2.0 (1.8)	
Exposure to FP discussions during intervention (%)						
Yes		60.9			57.9	

^a^We restricted our sample to women aged 15–45 and men 18–49 in both survey rounds, in order to compare women and men of the same age range at the two time points. Thus, only 590 of the 617 women who completed surveys at endline were included in our analysis, and only 285 of the 302 men who completed surveys at endline were included in our analysis. Some information was collected only at endline

^b^Chi-squared tests used to compare percentages and t-tests used to compare means

^c^Difference between age of male and female partners

^d^Std dev: Standard deviation

^e^Range 1–3

^f^Range 1–2

^g^Range 1–5

^h^Range 4–12

^i^Range 1–12

^j^Range 5–15

^k^Range 1–5

Women reported completing fewer years of education than men at both time points, and endline samples comprised slightly more educated men and women. About half of men and women in both samples reported being Protestant. At both interview times, about a third of women and three fifths of men reported working outside the home for cash. Fewer men and women at endline than at baseline reported that women made decisions about the cash they earned: 28.4% vs 35.4%, respectively, among men and 31.6% vs 37.8%, respectively, among women.

As measured at endline, men in this sample did not report strong support for traditional gender roles (mean score of 2.0 on a scale of 1–5, where 5 indicates stronger agreement with traditional gender roles). Both sexes reported that women had moderate to high household decision-making power (mean of 1.6 for both men and women on a scale ranging between 1 and 2, where a higher score indicates higher women’s decision-making power). Also, men and women both reported moderate to high levels of spousal communication (mean for women 2.9, and for men, 3.4, on a scale of 1–5 where 5 indicates high levels of spousal communication.)

At endline, only 11.6% of men as compared to 66.3% of women reported approving of women using family planning without their partners’ permission. Very few women (15.4%) believed they could suggest the use of condoms to their husbands. Men and women also reported widely incongruent pregnancy intentions: 30.2% of women interviewed at baseline and 35.1% at endline reported wanting to get pregnant at the time of their last pregnancy, but 60.0% and 64.2% of men reported so at baseline and endline, respectively. Notably, over 20% of women at both time points reported wanting to limit childbearing compared to only 4.3% and 8.4% of men at baseline and endline, respectively.

#### Changes in Family Planning Use from Baseline to Endline

Table 2 shows unmatched and propensity score-matched differences in use of any and of modern methods of family planning between baseline and endline for men and women. Based on propensity score-matched results, 36.5% and 34.0% of women used any method and a modern method, respectively, at baseline, while 51.8% and 51.2%, respectively, did so at endline. Similarly, 33.7% and 27.9% of men used any method and a modern method, respectively, at baseline, and 53.8% and 52.2% did so, respectively, at endline.

**Table 2:** Changes in family planning use between baseline and endline.

Outcome	Baseline (%)	Endline (%)	% difference (std dev)
**Women**			
**Current use of any method**			
Unmatched	31.7	51.7	20.0 (2.7)
Propensity score-matched^a^	36.5	51.8	15.3 (3.4)
**Current use of modern methods**			
Unmatched	29.4	51.0	21.6 (2.7)
Propensity score-matched	34.0	51.2	17.3 (3.2)
**Men**			
**Current use of any method**			
Unmatched	29.5	54.0	24.5 (4.0)
Propensity score-matched	33.7	53.8	20.1 (5.0)
**Current use of modern methods**			
Unmatched	24.3	52.6	28.3 (3.9)
Propensity score-matched	27.9	52.2	24.3 (4.8)

^a^Propensity scores (kernel matching technique) derived from logistic regression models with survey round (endline vs baseline) as outcome variable adjusted for the following covariates: respondent’s age, age difference between respondent and his/her partner, number of living children, education, partner’s education, whether the respondent works for cash, whether the woman makes decisions regarding spending of any cash earned, pregnancy intentions, the number of family planning methods known, the summed score on the index assessing family planning beliefs, the summed score on index assessing approval of family planning use, knowledge of where to obtain family planning (men only,) score on a scale assessing beliefs about gender roles (men only), score on family planning self-efficacy scale (women only) and whether they believed they could suggest the use of condoms to spouse (women only).

At baseline, 19.5% of women in the sample were using injectables, 3.3% condoms, 2.9% oral contraceptives, 2.6% had a tubal ligation, 1.4% used implants or IUDs, and 2.0% traditional methods. Notably different at endline, 29.7% of women in the sample were using injectables, 8% used oral contraceptives and 6.8% condoms. Among men, 18.5% and 27.2% reported use of condoms at baseline and endline, respectively; while 4.7% and 3.7% reported that their partners used injectables and oral contraceptives at baseline, 18.3% and 10.4% did so, respectively, at endline (data not shown).

#### Predictors of Family Planning at Endline

Our fully adjusted regressions models identified significant associations between participation in the intervention and both current use of any method and current use of a modern method of family planning for women (Table 3), but not for men (Table 4). As expected, the strongest associations were found among women exposed to discussions specifically about family planning: these women were 1.78 times more likely to use any method or a modern method than women who were not exposed to these discussions. Yet exposure to any topic covered during the intervention increased the odds of women using any method of family planning by 62% and the odds of using a modern method by 60%.

**Table 3:** Key determinants of and use of family planning among women at endline.

Characteristics	Current use any method					Current use modern method			
	Model I ^a^ OR^b^ (95% CI^c^)	Model II ^a^ OR (95% CI)		Model III ^a^ OR (95% CI)		Model I ^a^ OR (95% CI)	Model II ^a^ OR (95% CI)		Model III ^a^ OR (95% CI)
**Socio-demographic**									
Age (years; <25 = ref)									
25–29	**1.89 (1.15, 3.10)**		**1.89 (1.15, 3.11)**		**1.86 (1.13, 3.06)**	**1.84 (1.12, 3.01)**		**1.85 (1.13, 3.03)**	**1.81 (1.11, 2.97)**
30–34	**1.82 (1.01, 3.27)**		**1.80 (1.00, 3.24)**		**1.83 (1.01, 3.29)**	**1.87 (1.04, 3.34)**		**1.86 (1.04, 3.33)**	**1.88 (1.05, 3.37)**
≥35	0.79 (0.40, 1.57)		0.77 (0.39, 1.53)		0.78 (0.39, 1.56)	0.78 (0.39, 1.53)		0.76 (0.38, 1.50)	0.77 (0.39, 1.53)
Age difference between partners (years; <5 = ref)^d^									
5–9	0.99 (0.62, 1.56)		1.02 (0.64, 1.62)		0.99 (0.62, 1.57)	0.91 (0.57, 1.44)		0.94 (0.59, 1.49)	0.91 (0.57, 1.43)
10–14	1.12 (0.65, 1.91)		1.13 (0.66, 1.93)		1.11 (0.65, 1.89)	1.10 (0.65, 1.88)		1.12 (0.66, 1.91)	1.09 (0.64, 1.86)
≥15	1.02 (0.56, 1.85)		1.02 (0.56, 1.86)		1.02 (0.56, 1.85)	1.00 (0.55, 1.81)		1.01 (0.56, 1.84)	1.00 (0.55, 1.82)
Number of living children									
	**1.21 (1.06, 1.37)**		**1.21 (1.07, 1.38)**		**1.20 (1.05, 1.36)**	**1.20 (1.06, 1.36)**		**1.21 (1.06, 1.37)**	**1.19 (1.05, 1.36)**
Religion (Christian Protestant = ref)^e^									
Catholic	0.89 (0.57, 1.30)		0.88 (0.56, 1.38)		0.90 (0.57, 1.41)	0.91 (0.58, 1.42)		0.90 (0.58, 1.41)	0.92 (0.59, 1.44)
Other	0.88 (0.55, 1.42)		0.87 (0.54, 1.39)		0.90 (0.56, 1.44)	0.90 (0.57, 1.44)		0.89 (0.56, 1.42)	0.92 (0.57, 1.47)
Education (years)									
	1.00 (0.94, 1.07)		1.00 (0.94, 1.07)		0.99 (0.93, 1.06)	0.99 (0.93, 1.06)		1.00 (0.93, 1.07)	0.99 (0.92, 1.06)
Partner education (years)									
	1.05 (0.99, 1.12)		1.05 (0.98, 1.11)		1.05 (0.98,1.11)	1.04 (0.98, 1.11)		1.04 (0.98, 1.10)	1.04 (0.98, 1.10)
**Women’s empowerment / Gender beliefs /Relationship with partner/pregnancy intentions**									
Woman makes decisions about spending the cash she earns (women with no cash earned = ref)^e^									
All cash	1.46 (0.91, 2.33)		1.50 (0.93, 2.39)		1.49 (0.93, 2.38)	**1.59 (1.00, 2.54)**		**1.63 (1.02, 2.60)**	**1.62 (1.02, 2.59)**
Some cash	1.27 (0.67, 2.40)		1.22 (0.64, 2.31)		1.29 (0.68, 2.44)	1.24 (0.66, 2.32)		1.19 (0.63, 2.23)	1.26 (0.67, 2.36)
Participation in household decision-making scale score									
	0.71 (0.28, 1.79)		0.72 (0.29, 1.79)		0.72 (0.29, 1.81)	0.66 (0.26, 1.63)		0.65 (0.26, 1.63)	0.66 (0.26, 1.65)
Inter-spousal communication scale									
	**1.32 (1.05, 1.65)**		**1.30 (1.04, 1.63)**		**1.30 (1.04, 1.64)**	**1.30 (1.04, 1.63)**		**1.29 (1.03, 1.61)**	**1.29 (1.03, 1.61)**
Pregnancy intentions (wants pregnancy in next 12 months at time of survey = ref)									
Wants to delay pregnancy for ≥ 1 year	0.91 (0.57, 1.47)		0.93 (0.58, 1.50)		0.90 (0.56, 1.45)	0.93 (0.58, 1.49)		0.95 (0.59, 1.52)	0.91 (0.57, 1.47)
Wants no more children	0.98 (0.57, 1.67)		0.98 (0.57, 1.69)		0.96 (0.56, 1.66)	1.01 (0.59, 1.72)		1.01 (0.59, 1.72)	0.99 (0.58, 1.69)
Has not thought about/not sure/not applicable	0.64 (0.37, 1.13)		0.62 (0.35, 1.09)		0.62 (0.35, 1.09)	0.66 (0.38, 1.15)		0.63 (0.36, 1.11)	0.63 (0.36, 1.10)
**Family planning knowledge/attitudes/subjective norms self-efficacy**									
Family planning beliefs score									
	0.99 (0.90, 1.10)		0.99 (0.89, 1.09)		0.99 (0.90, 1.09)	0.98 (0.89, 1.09)		0.98 (0.89, 1.08)	0.98 (0.89, 1.08)
# methods known									
	1.10 (0.95, 1.26)		1.11 (0.96, 1.27)		1.09 (0.95, 1.26)	1.05 (0.91, 1.20)		1.06 (0.92, 1.21)	1.04 (0.91, 1.20)
Woman can use family planning without partner’s permission (no = ref)									
	*1*.*48 (0*.*98*, *2*.*21)*		*1*.*50 (0*.*99*, *2*.*25)*		*1*.*50 (0*.*99*, *2*.*25)*	*1*.*45 (0*.*97*, *2*.*18)*		*1*.*48 (0*.*99*, *2*.*21)*	*1*.*47 (0*.*98*, *2*.*21)*
Woman can suggest use of condoms (disagree/unsure = ref)									
	0.96 (0.56, 1.65)		0.96 (0.56, 1.65)		1.00 (0.58, 1.72)	0.98 (0.57, 1.68)		0.97 (0.57, 1.67)	1.02 (0.59, 1.74)
Score on family planning use approval questions									
	0.99 (0.89, 1.09)		0.99 (0.89, 1.09)		0.99 (0.89, 1.09)	0.99 (0.89, 1.09)		0.99 (0.89, 1.09)	0.99 (0.89, 1.09)
Family planning self-efficacy scale score									
	**1.39 (1.12, 1.73)**		**1.40 (1.12, 1.74)**		**1.36 (1.09, 1.69)**	**1.39 (1.12, 1.72)**		**1.39 (1.12, 1.72)**	**1.35 (1.09, 1.68)**
**Exposure to intervention**									
Exposure to intervention (no = ref)	**1.62 (1.08, 2.41)**					**1.60 (1.07, 2.38)**			
Number of topics exposed to during intervention			**1.15 (1.02, 1.30)**					**1.16 (1.02, 1.31)**	
Exposure to FP discussions during intervention (no = ref)					**1.78 (1.20, 2.65)**				**1.78 (1.20, 2.63)**

^a^ Models adjusted for all factors shown; Model I adjusted for exposure to the intervention (yes/no); Model II adjusted for the number of topics of exposure; Model III adjusted for exposure to FP discussions

^b^OR: Odds ratio: statistically significant ORs at p<0.05 are shown in bold; statistically significant ORs at p<0.10 are shown in italic.

^c^CI: Confidence Interval

^d^Difference between age of male and female partners

^e^Category for missing data indicator included in the model.

**Table 4:** Key determinants of FP use among men at endline.

Characteristics	Current use any method			Current use modern method		
	Model I^a^ OR^b^ (95% CI)^c^	Model II^a^ OR (95% CI)	Model III^a^ OR (95% CI)	Model I^a^ OR (95% CI)	Model II^a^ OR (95% CI)	Model III^a^ OR (95% CI)
**Socio-demographic**						
Age (years; <25 = ref)						
25–29	0.56 (0.22, 1.41)	0.55 (0.22, 1.39)	0.56 (0.22, 1.42)	0.64 (0.26, 1.59)	0.63 (0.25, 1.57)	0.64 (0.26, 1.60)
30–34	0.87 (0.32, 2.34)	0.85 (0.31, 2.29)	0.88 (0.33, 2.36)	0.99 (0.37, 2.64)	0.97 (0.36, 2.59)	1.00 (0.37, 2.65)
≥35	0.82 (0.26, 2.63)	0.81 (0.26, 2.58)	0.83 (0.26, 2.65)	0.92 (0.29, 2.90)	0.92 (0.29, 2.87)	0.93 (0.30, 2.93)
Age difference between partners (years; <5 = ref)^d^						
5–9	**2.72 (1.39, 5.32)**	**2.79 (1.42, 5.49)**	**2.71 (1.38, 4.31)**	**2.62 (1.35, 4.10)**	**2.68 (1.37, 4.22)**	**2.62 (1.35, 4.09)**
10–14	**2.82 (1.06, 5.53)**	**2.81 (1.05, 6.51)**	**2.82 (1.05, 4.52)**	2.15 (0.82, 4.49)	2.13 (0.82, 4.57)	2.14 (0.82, 4.49)
≥15	2.32 (0.53, 6.04)	2.24 (0.52, 5.68)	2.21 (0.51, 5.51)	2.45 (0.57, 5.98)	2.38 (0.56, 5.90)	2.37 (0.55, 5.87)
Number of living children						
	0.99 (0.82, 1.20)	0.99 (0.82, 1.20)	0.99 (0.82, 1.20)	1.00 (0.82, 1.20)	1.00 (0.82, 1.20)	1.00 (0.82, 1.21)
Religion (Christian Protestant = ref)^e^						
Catholic	1.29 (0.66, 2.50)	1.29 (0.67, 2.51)	1.28 (0.66, 2.47)	1.27 (0.66, 2.45)	1.27 (0.66, 2.46)	1.25 (0.65, 2.41)
Other	1.79 (0.80, 3.99)	1.88 (0.83, 3.22)	1.72 (0.80, 3.58)	1.49 (0.67, 3.30)	1.55 (0.70, 3.47)	1.48 (0.67, 3.27)
Education (years)						
	1.04 (0.94, 1.16)	1.04 (0.94, 1.16)	1.04 (0.94, 1.16)	1.03 (0.92, 1.15)	1.03 (0.92, 1.15)	1.03 (0.92, 1.15)
Partner education (years)						
	*1*.*12 (0*.*98*, *1*.*29)*	*1*.*13 (0*.*98*, *1*.*29)*	*1*.*12 (0*.*98*, *1*.*28)*	1.12 (0.97, 1.28)	1.12 (0.98, 1.29)	1.11 (0.97, 1.28)
Man works outside home for cash (no = ref)						
	1.64 (0.86, 3.13)	1.65 (0.86, 3.16)	1.65 (0.87, 3.15)	1.68 (0.89, 3.19)	1.69 (0.89, 3.21)	1.69 (0.89, 3.20)
**Gender beliefs and roles /relationship with partner/pregnancy intentions**						
Woman makes decisions re spending cash she earns (women with no cash earned = ref)^e^						
All cash	1.85 (0.74, 4.34)	1.80 (0.72, 3.51)	1.83 (0.73, 3.59)	1.91 (0.77, 3.76)	1.87 (0.75, 3.33)	0.90 (0.76, 3.72)
Some cash	1.02 (0.36, 2.87)	0.69 (0.27, 1.82)	0.72 (0.28, 1.89)	0.55 (0.21, 1.41)	0.54 (0.21, 1.40)	0.56 (0.22, 1.45)
Male dominance scale score						
	**0.55 (0.31, 0.98)**	**0.52 (0.29, 0.94)**	**0.54 (0.30, 0.97)**	**0.57 (0.32, 1.00)**	**0.55 (0.31, 0.98)**	**0.57 (0.32, 1.00)**
Participation in household decision-making scale score						
	1.69 (0.45, 3.38)	1.69 (0.45, 4.38)	1.71 (0.45, 4.04)	1.77 (0.47, 4.15	1.76 (0.47, 4.13)	1.78 (0.47, 4.18)
Inter-spousal communication scale						
	1.08 (0.74, 1.59)	1.06 (0.72, 1.56)	1.08 (0.74, 1.59)	1.08 (0.74, 1.59)	1.07 (0.73, 1.56)	1.09 (0.74, 1.59)
Pregnancy intentions (wants pregnancy in next 12 months at time of survey = ref)						
Wants to delay pregnancy for ≥ 1 year	1.63 (0.61, 3.35)	1.70 (0.63, 3.55)	1.58 (0.59, 3.26)	1.78 (0.67, 3.73)	1.85 (0.70, 3.90)	1.75 (0.66, 3.68)
Wants no more children	**3.19 (1.04, 6.74)**	**3.34 (1.08, 7.27)**	**3.30 (1.08, 7.10)**	2.58 (0.87, 5.43)	2.66 (0.89, 5.60)	2.64 (0.90, 5.60)
Has not thought about/not sure/not applicable	0.97 (0.43, 2.19)	0.97 (0.43, 2.21)	0.96 (0.42, 2.18)	1.06 (0.47, 2.38)	1.06 (0.47, 2.40)	1.06 (0.47, 2.38)
**Family planning knowledge/attitudes/subjective norms self-efficacy**						
Family planning beliefs score						
	**1.16 (1.00, 1.36)**	**1.17 (1.00, 1.36)**	**1.16 (1.00, 1.36)**	1.13 (0.97, 1.31)	1.13 (0.97, 1.31)	1.12 (0.97, 1.31)
# methods known						
	1.02 (0.85, 1.23)	1.00 (0.82, 1.21)	1.01 (0.84, 1.22)	1.01 (0.84, 1.22)	0.99 (0.82, 1.20)	1.01 (0.84, 1.21)
Woman can use family planning without partner’s permission						
	1.48 (0.60, 3.63)	1.50 (0.61, 3.65)	1.43 (0.59, 3.46)	1.30 (0.52, 3.19)	1.31 (0.54, 3.18)	1.26 (0.52, 3.04)
Score on family planning use approval questions						
	**1.53 (1.25, 1.88)**	**1.53 (1.25, 1.89)**	**1.53 (1.24, 1.87)**	**1.45 (1.19, 1.77)**	**1.45 (1.19, 1.77)**	**1.44 (1.18, 1.76)**
Knows where to get family planning (no = ref)						
	0.34 (0.06, 1.85)	0.34 (0.06, 1.84)	0.34 (0.06, 1.86)	0.15 (0.02, 1.36)	0.15 (0.02, 1.35)	0.15 (0.02, 1.36)
**Exposure to intervention**						
Exposure to intervention (no = ref)						
	1.26 (0.67, 2.40)			1.22 (0.65, 2.30)		
Number of topics of exposed to during intervention						
		1.12 (0.94, 1.33)			1.10 (0.93, 1.30)	
Exposure to family planning discussions during intervention (no = ref)						
			1.32 (0.72, 2.40)			1.23 (0.68, 2.23)

^a^ Models adjusted for all factors shown; Model I adjusted for exposure to the intervention (yes/no); Model II adjusted for the number of topics of exposure; Model III adjusted for exposure to FP discussions

^b^OR: Odds ratio: statistically significant ORs at p<0.05 are shown in bold; statistically significant ORs at p<0.10 are shown in italic.

^c^CI: Confidence interval

^d^Difference between age of male and female partners

^e^Category for missing data indicator included in the model

We also found that women were significantly more likely to report use of any method or of a modern method of family planning if they reported more spousal communication and higher self-efficacy to use family planning. Reported control over their own cash earnings was significantly associated with women’s use of modern methods. Beliefs that women can use family planning without their spouses’ permission also appears to be positively associated with current use of any or of modern methods, but these associations were not statistically significant (at p<0.05). Women’s younger age and higher number of living children were also significant positive predictors of use of both any and modern family planning methods.

As noted, none of the intervention exposure measures was significantly associated with men’s reports of current use of any or modern methods of family planning. However, men were significantly more likely to use any or a modern method if they had higher approval scores for use of family planning. Further, for both any and modern methods, men who reported more traditional beliefs about gender roles were less likely to use family planning. Men who reported accurate knowledge about family planning were more likely to use modern methods, but this relationship was not statistically significant. Finally, men who reported that they did not want to have any more children were three times as likely to use any method as those who wanted a pregnancy in the future.

### Qualitative Results

#### Description of the Sample

Data for this analysis were drawn from ten married couples. All couples were determined to be ‘real’ marriages or current unions based on the triangulation of key events and stories in the relationship histories from both partners. All couples reported living together in Siaya County during the intervention timeframe. At the time of data collection, couples had been married for between 5 and 32 years. Women were between the ages of 21 and 49 years; four were over the age of 35. Men’s ages ranged between 23 and 68 years. Wives were younger than their husbands in all of the relationships, and in six of the 10 couples spouses were within 5 years of age of each other.

All couples reported a pregnancy within the first two years of marriage, and at least half of the women reported becoming pregnant prior to marriage. Seven of the ten women interviewed reported having dropped out of school due to pregnancy, lack of school fees, or both. Women reported having between 2 and 5 living children, and one was pregnant at the time of the interview. All respondents reported using at least one method of family planning at some point during their marriage, including calendar, condoms, pills, IUD, injections, implants, and bilateral tubal ligation. Five women reported using a family planning method in secret at some point during their marriage. Two of the relationships were polygamous, and in five relationships the husband was known or suspected to have had sex with someone else during the marriage. There were no reports of wives having other sexual partners than their husbands during the marriage. All couples reported one or both partners having been tested for HIV. In five couples, one or both partners reported incidents of inter-partner violence, all of which involved husbands physically abusing their wives.

There was variability in how couples were introduced to the intervention. In some cases, initial exposure occurred by accident (encountered a drama group performing in the community) and in others by invitation (a friend invited them to join a group for couples). Likewise, sometimes the husband participated in dialogues, sometimes the wife participated, and sometimes both husband and wife from the same couple participated in the same dialogues. Men and women from two of the couples interviewed reported actively sharing their stories about family planning during a dialogue convened through the intervention.

#### Experience of Participation in the Intervention

Several participants discussed attending dialogues about family planning at *barazzas* (community meetings) or events.

*“I was invited to these meetings by a friend*, *and during these meetings there were discussions about role-sharing at home and also [use of] family planning*!*”*

Men and women reported learning about family planning in community dialogues. One woman recalled her husband’s report upon returning from a *barazza*.

*“Today the discussion at the Chief’s* barazza *was very heated*. *Imagine*, *we discussed about family chores and family planning*. *I have never known that family planning issues were real*.*”*

The intervention offered exposure to discussion of the benefits of family planning: one woman recounted how her peers not only described, but provided ‘living proof,’ of positive experiences. Both men and women reported learning by contrasting real examples of health and economic stability between family planning users and non-users in the community. Others described feeling comfortable publicly sharing their own family planning stories when they perceived that the social environment was becoming more openly supportive of couples’ use of family planning. One man, who had previously opposed family planning, described how learning of others’ positive experiences dispelled his beliefs that it caused infertility and birth defects. Dialogues appear to have increased the acceptability of not only talking about, but also using family planning, especially when key opinion leaders (e.g. chiefs and religious leaders) both advocated for and modeled open communication with spouses and use of family planning. One woman described how community members openly supported others to use family planning during the dialogues; she and her husband reported that their ongoing involvement in dialogues about family planning and gender roles influenced how they communicated about these issues at home.

#### Shifting Gender Norms

Women described shifts towards more equitable household roles, with husbands beginning to help with household duties. Some men also described helping with chores that were traditionally considered ‘women’s work.’ For example, one man spoke of learning the importance of sharing household responsibilities at a dialogue. He cited collecting firewood, going to the *posho* mill, cooking and fetching water as examples of things he now does. One woman relayed a story of how she and the children were shocked when, unprompted, her husband cooked for the family. She recalled her children telling her:

*“Mom*! *Guess what happened in our house today? Dad cooked us lunch!”*

Some couples described starting to manage household budgets together and making more joint decisions about household purchases and assets. One woman said that one of the best moments of her marriage was when she and her husband “*made decisions together as man and wife*.” Together, they decided to sell the scrap metal her husband had accumulated and bought a cow; when the cow was old enough, they sold it and bought materials for the new house they are now planning to build.

One man said:

*“Before*, *what was ours was mine*. *Now*, *what’s ours is ours*. *“*

Despite such reported changes, both men and women stated that women still did the majority of household work, and that men maintained the majority of household decision-making power.

#### Couple Communication

Men and women reported more open and equitable communication about family planning. Several women who had previously used a method in secret reported less opposition towards family planning from their partners, and described how they now openly communicated and reached mutual agreement to use a method. One man reported that he reminded his wife to get regular injections and offered to accompany her to the clinic. Another man described extensive communication on selecting a family planning method: the couple jointly decided against oral contraceptives because of the need for daily adherence, and instead chose injectable contraception.

Men reported that couple concordance about fertility intentions and desired family size helped them resist external pressure for large families. Previously, some women had experienced conflict about the ideal number of children and family planning use, including questioning and pressure from husbands about getting pregnant. Several men and women reported that improved communication with their spouses both decreased conflict about family planning and increased the “*love and harmony*” in their relationship overall.

One woman cited her husband’s support for family planning as an enabler for her use of family planning: it boosted her morale and brought harmony to her marriage. Previously she had used family planning without her husband’s consent or knowledge, and this had contributed stress and conflict in the relationship.

While women described the many benefits of having partner support for using family planning, including increased relationship harmony, they also discussed the critical importance of being able to independently access family planning methods when faced with partner opposition.

One woman recalled using family planning without her husbands’ permission as a time when she was able to control something about the relationship, and was happy to be free of worry that she would become pregnant.

Another recalled that she had learned about family planning methods in the privacy of a health care facility, and started using a method in secret. Family planning use, she said, had helped ensure her children were adequately spaced and that she had children when she had the resources to support them, but she also described experiencing significant stress because her husband continued to pressure her for a baby and was suspicious when she did not get pregnant. She also spoke of the stress of managing side effects on her own. Subsequently, she and her husband came to agreement about the use of family planning, and she has recently switched to a more effective method with her husband’s support. However, she stated that she would still be using a method in secret if her husband had not changed his attitude. She emphasized that her previous use of family planning had “*cost her harmony…but it was worth it*” to be able to control her fertility.

## Discussion

Our results suggest that an intervention that supports open and public dialogue about gender, sexuality, and family planning may increase use of family planning among couples in settings where the prevalence of use is relatively low. First, self-reported family planning use increased significantly for both women and men in our study samples between baseline and endline. Reports of family planning use by women at baseline closely matched use estimates for Nyanza Province in the 2008–2009 Kenya DHS [21]. In the analysis of endline data, use of family planning by women was significantly associated with participation in the intervention, particularly in specific dialogues on family planning. The intervention’s effects were significant for use of both any and modern contraceptive methods and after adjusting for key socio-demographic factors. While similar interventions may have been implemented in similar settings, there are limited evaluation data of such interventions in the published literature. Yet, in line with results from our study, published evaluation data show positive effects of similar community-level interventions in addressing social factors and shifting social norms with regard to reproductive health issues. For example, in 2004, CARE implemented and evaluated a 3-year adolescent reproductive health project to address related behavior changes and local social norms in a rural district of the Republic of Georgia. Community engagement strategies included promoting community support for adolescent reproductive health and using 'Theatre for Development' to promote community dialogue about social norms. Project evaluation data demonstrated improved knowledge, attitudes, behavior about FP and evidence of shifts in gender norms. [25] More recently, Campbell et al. explored pathways between community participation and HIV prevention, treatment and impact mitigation in Zimbabwe, reviewing six qualitative studies in Manicaland [26]. These found that community group membership is often associated with decreased HIV incidence and that participation in formal community groups and informal local networks provides opportunities for critical dialogue about HIV/AIDS, often facilitating renegotiation of harmful social norms, sharing of personal experiences of HIV/AIDS, formulation of positive action plans and solidarity to action them.

We found that a greater level of spousal communication, and higher self-efficacy to discuss and use family planning, were significant predictors of use for women at endline. As our theory of change suggests, couple communication and self-efficacy are two of three key variables we expected to be positively associated with exposure to the intervention. However, the third key variable—women’s greater participation in household decision-making—was not a significant predictor of family planning use. The comprehensive household decision-making measure we used assessed decisions across a wide range of domains. While it may provide a more robust measure of women’s overall decision-making power in the household, it is not narrowly focused on decisions about family size, contraceptive use, or healthcare seeking, and therefore may be less predictive of such outcomes; future studies could compare various proposed measures of household decision-making power to compare their predictive power on a range of maternal health outcomes, including family planning. Moreover, it may be that self-efficacy to use family planning is a better measure of women’s power to enact these behaviors, and may have accounted for most of the variance in this domain. Reported control over their own cash earnings was also significantly associated with use of modern methods, suggesting that economic empowerment may be associated with access to and/or use of FP. Finally, greater age and a higher number of children were significantly associated with women’s family planning use, yet women’s reproductive intentions were not a significant predictor of use.

For men, approval of family planning, more equitable (less traditional) beliefs about gender roles, and a desire to not have more children were the strongest predictors of family planning use. Neither the scale measuring women’s decision-making power in the household, nor any of the intervention exposure variables, were significant predictors of men’s current use of any or of modern methods.

Of note, the two areas with the most striking differences in men’s and women’s responses at endline were reproductive intentions (60.0% of men and 30.2% of women wanted another pregnancy at the time of the interview or within the next 12 months) and approval of women’s use of family planning without her partners’ permission (66.3% of women and only 11.6% of men approved of such use). Given these differences between men and women, an intervention that supports more open communication about reproductive decision-making and family planning seems appropriate. Our findings suggest that improved communication may have enabled more couple acceptance of family planning, and thus increased use of family planning over time. In addition, men’s desire not to have any more children was a strong predictor of family planning use among men, whereas women’s reproductive intentions were not a predictor of family planning use among women even though women were much more likely to want to delay or limit childbearing than were men. Rather than indicate that men’s childbearing desires are more important to a couple’s use of family planning use than women’s, these results might suggest that when men’s intentions are more closely aligned with women’s, the couple is more likely to use family planning.

Our qualitative results provide additional insight into the ways in which the intervention may have contributed to changes in couple communication and family planning behavior–the mixed-method approach we used is one of the strengths of our analysis. Public dialogues may have increased the perceived social acceptability of family planning and of the benefits of open couple communication about family planning, and these normative shifts at community level may have enabled more equitable communication and decision-making at the couple level. Ongoing, public discourse—often explicitly supported or led by community leaders—may have normalized discussions about family planning and influenced family planning acceptability at the community level. The participation of prominent male opinion leaders in these dialogues may have helped to legitimize men’s participation in communication and decision-making about family planning, and may have positively influenced their approval of family planning, thus increasing use. Our quantitative results support this: men’s approval of family was an important predictor of family planning use.

Public discourse precipitated conversations on family planning in the household as couples discussed what they heard and learned. Men described a change in their acceptance of family planning, and women reported that men began to demonstrate more shared decision-making, better communication, and increased support for family planning. Male support of and male involvement in family planning also appear to be important factors for helping with method adherence. Women reported partners accompanying them to get methods and reminding them to take their pills daily. Increased couple communication may also have contributed to increased harmony and a decrease in conflict. Relationship harmony was highly valued by both men and women.

## Limitations

Our survey data are cross-sectional without a comparison group and we used a two-stage cluster sampling approach, thus the sample obtained does not cover the population as evenly as in the case of simple random sampling. Both our quantitative and qualitative results are subject to self-report bias. Propensity scores were used to reduce selection bias by equating the baseline and endline survey samples based on key measured covariates, yet propensity score matching only accounts for observed covariates: imbalances may remain even after propensity score adjustment if relevant survey respondent characteristics were not measured or were measured imprecisely. Thus, the larger estimated differences in baseline-endline family planning use based on unmatched results may be real. Importantly, self-selection to participation in the intervention is a limitation of our analyses examining associations between exposure to the intervention and family planning use at endline. Yet, some reassurance is provided since the intervention was widely advertised and supported by community leaders, and since 65% of the respondents were exposed. In addition, we cannot account for potential effects of other sources of family planning information (e.g. health providers, HIV/STI testing and counseling, domestic violence counseling, media) to the observed differences in family planning use between baseline and endline surveys and the associations observed at endline. However, to our knowledge, no other similar interventions took place in the study area during the intervention period. Also, the intensity of the intervention was measured as the number of topics covered during intervention activities attended and not as the amount of time of exposure to the intervention. Our qualitative interview guide elicited more reports on the positive than the negative aspects of the intervention and family planning use; this may be the result of social desirability bias or of the nature of our intervention activities (i.e. involvement of community leaders, theatre groups, other forms of entertainment). Finally, we do not have information on women who refused to be interviewed or completed only part of the questionnaire.

## Conclusion

Our results suggest that an intervention that encourages and supports dialogue and communication about gender norms and sexuality can shift gender relations and positively influence family planning use, especially for women. Participation in the intervention and greater spousal communication, in addition to variables indicative of more empowerment (self-efficacy for family planning and control over household assets), all contributed to greater use of family planning for women. For men, approval of family planning, more equitable beliefs about gender roles, and a desire to not have more children were the strongest predictors of family planning use. These findings are consistent with other research that suggests more equitable gender norms and spousal communication are linked to contraceptive use and positive reproductive health outcomes, and provide an effective intervention model for achieving those goals through community-based dialogues. While not definitive, our promising evaluation results now allow for smaller scale studies to examine the impact of specific types of dialogues and experience sharing on specific outcomes. Future studies should consider the local context and, if possible, use a similar mixed-method approach.

## Supporting Information

S1 File WE MEASR Scales and Indices. (DOC)

S2 File Family Planning Measures. (DOC)

S3 File Men’s Data. (XLS)

S4 File Women’s Data. (XLSX)

S5 File Codebook for Women’s and Men’s Datasets. (DOC)
