# Supplementary material for: Community Dialogue to Shift Social Norms and Enable Family Planning: An Evaluation of the Family Planning Results Initiative in Kenya
Source: PLoS One. 2016 Apr 28;11(4):e0153907. doi: 10.1371/journal.pone.0153907 (PMC4849797; doi:10.1371/journal.pone.0153907)
Supplement: S1 File — (DOC) [file pone.0153907.s001.doc]

**CARE’s WE-MEASR Scales and Indices (Women’s Empowerment – Multidimensional Evaluation of Agency, Social Capital and Relations)**

**Support for Traditional Gender Roles (Male Dominance) (used with men only)**

*(Interviewer) I am now going to read some statements about relationships between men and women. Please tell me if you strongly agree, agree, neither agree nor disagree, disagree or strongly disagree.*

1. Changing diapers, giving the kids a bath, and feeding the kids are the mothers’ responsibility.
2. A man is the one who decides when to have sex with his wife.
3. Only when a woman has a child is she a real woman.
4. If a woman wants to avoid being pregnant, it is her responsibility alone.
5. It’s better to have more sons than daughters in a family.
6. Women have the same rights as men to work and study outside of their home.
7. A couple should decide together how many children to have
8. Men should help with the household duties
9. It is just as important for a girl to go to school as it is for a boy to go to school.

Item response options: 5-point Likert scale, where Strongly Agree = 5, Agree = 4, Neither Agree Nor Disagree = 3, Disagree = 2, and Strongly Disagree = 1

The scale was constructed by summing the item scores and dividing by the number of items. The scale score range is 1-5, and a higher scale score indicates a higher support for traditional gender roles (male dominance).

| **Sample** | **# of Items** | **Chronbach’s alpha** | **Mean** | **Standard Deviation** |
| --- | --- | --- | --- | --- |
| 317 men aged 18-49 | 9 | 0.66 | 3.21 | .44 |

References

Adapted with permission from: Pulerwitz J, Barker G. Measuring attitudes toward gender norms among young men in Brazil: Development and psychometric evaluation of the GEM Scale. Men and Masculinities. 2008; 10, 322-338.

**Interspousal Communication (used with women and men)**

*(Interviewer) I would now like to ask you about things you may discuss with your husband. For each one please tell me if you discuss this always, often, sometimes, seldom or never.*

1. *How often do you and your spouse discuss things that happened during the day?*
2. *How often do you and your spouse discuss your worries or feelings?*
3. *How often do you and your spouse discuss what to spend household money on?*
4. *How often do you and your spouse discuss when to have children?*
5. *How often do you and your spouse discuss whether to use family planning?*

Item response options: 5-point Likert scale, where Always = 5, Often = 4, Sometimes = 3, Seldom = 2 and Never = 1

The scale was constructed by summing the item scores and dividing by the number of items. The scale score range is 1-5, and a higher scale score indicates a higher level of interspousal communication.

| **Sample** | **# of items** | **Chronbach’s alpha** | **Mean** | **Std Dev** |
| --- | --- | --- | --- | --- |
| 302 men aged 18-49 | 5 | 0.63 | 1.63 | 0.25 |
| 617 women aged 18-45 | 5 | 0.75 | 2.94 | 0.94 |

References

Adapted with permission from: ICRW. (2011). Towards improved economic and sexual reproductive health outcomes for adolescent girls (TESFA). Baseline survey. Washington, DC: Author.

**Women’s Participation in Household Decision-making (used with women)**

*(Interviewer) Now I would like to ask you about who usually makes decisions in your household.*

1. *Which member of your household usually makes decisions about your health care?*
2. *Which member of your household usually makes decisions about making large household purchases?*
3. *Which member of your household usually makes decisions about making household purchases for daily needs?*
4. *Which member of your household usually makes decisions about when you will visit family/relatives/friends?*
5. *Which member of your household usually makes decisions about when your whole household will visit family/relatives/friends?*
6. *Which member of your household usually makes decisions about how to use the money that you bring into the household?*
7. *Which member of your household usually makes decisions about how to use the money your spouse brings into the household?*
8. *Which member of your household usually makes decisions about when your family will sell a large asset (like a cow)?*
9. *Which member of your household usually makes decisions about when your family will sell a small asset (like a chicken)?*
10. *Which member of your household usually makes decisions about whether you can work to earn money?*
11. *Which member of your household usually makes decisions about when you and your husband have sex?*
12. *Which member of your household usually makes decisions about whether you and your husband use family planning?*

Item response options: You, Your Husband, You and Your Husband Together, Mother- or Father-in-law, Someone Else, or Mother or Father. You or You and Your Husband Together = 2 and all other responses = 1

The scale was constructed by summing the item scores and dividing by the number of items. The scale score range is 1-2, and a higher scale score indicates more equitable decision-making in the household.

| **Sample** | **# of items** | **Cronbach's alpha** | **Mean** | **Standard Deviation** |
| --- | --- | --- | --- | --- |
| 583 women aged 18-45 | 12 | 0.74 | 1.62 | 0.23 |

**Women’s Participation in Household Decision-making (used with men)**

*(Interviewer) Now I would like to ask you about who usually makes decisions in your household.*

1. *Which member of your household usually makes decisions about making large household purchases?*
2. *Which member of your household usually makes decisions about making household purchases for daily needs?*
3. *Which member of your household usually makes decisions about when you will visit family/relatives/friends?*
4. *Which member of your household usually makes decisions about when your whole household will visit family/relatives/friends?*
5. *Which member of your household usually makes decisions about how to use the money that you bring into the household?*
6. *Which member of your household usually makes decisions about how to use the money your spouse brings into the household?*
7. *Which member of your household usually makes decisions about when your family will sell a large asset (like a cow)?*
8. *Which member of your household usually makes decision about when your family will sell a small asset (like a chicken)?*
9. *Which member of your household usually makes decisions about when you and your wife have sex?*
10. *Which member of your household usually makes decisions about whether you and your husband use family planning?*

Item response options: You (husband), Your Wife, You and Your Wife Together, Mother- or Father-in-law, Someone Else, or Mother or Father. Your Wife or You and Your Wife Together = 2 and all other responses = 1

The scale was constructed by summing the item scores and dividing by the number of items. The scale score range is 1-2, and a higher scale score indicates more equitable decision-making in the household.

| **Sample** | **# of items** | **Chronbach’s alpha** | **Mean** | **Standard Deviation** |
| --- | --- | --- | --- | --- |
| 302 men aged 18-49 | 10 | 0.79 | 1.63 | 0.25 |

References

Adapted with permission from: Pulerwitz J, Gortmaker SL, Dejong W. Measuring relationship power in HIV/STD research. Sex Roles. 2000; 42 (7&8), 637‐660. 3.; and

MEASURE DHS & ICF Macro. (2008). *Demographic and health survey (DHS)*. Calverton, MD: Author.

Self-efficacy to Discuss and Use Family Planning (used with women only)

*(Interviewer) Now I am going to ask some questions about how confident or sure you are that you could use family planning if you wanted to. Even if you do not want to use family planning right now, try to imagine sometime in the future when you might wish to use family planning.*

1. *How sure are you that you could bring up the topic of family planning with your husband?*
2. *How sure are you that you could tell your husband that you wanted to use family planning?*
3. *How sure are you that you could use family planning?*
4. *How sure are you that you could use family planning, even if your husband did not want to?*

Item response options: 5-point Likert scale, where Completely Sure = 5, Somewhat Sure = 4, Neither Sure/Unsure = 3, Somewhat Unsure = 2, and Not at all Sure = 1

The scale was constructed by summing the item scores and dividing by the number of items. The scale score range is 1-5, and a higher scale score indicates higher self-efficacy to discuss and use family planning.

| **Sample** | **# of Items** | **Cronbach's**  **alpha** | **Mean** | **Standard Deviation** |
| --- | --- | --- | --- | --- |
| 616 women aged 18-45 | 4 | 0.72 | 4.16 | 1.03 |
