# Supplementary material for: Community Dialogue to Shift Social Norms and Enable Family Planning: An Evaluation of the Family Planning Results Initiative in Kenya
Source: PLoS One. 2016 Apr 28;11(4):e0153907. doi: 10.1371/journal.pone.0153907 (PMC4849797; doi:10.1371/journal.pone.0153907)
Supplement: S2 File — (DOC) [file pone.0153907.s002.doc]

**Family Planning Indices**

**Family Planning Beliefs Index** (used with men and women)

*(Interviewer) “Now I would like to read you some statements about family planning methods. For each one I'd like you to tell me whether you agree, disagree, or are unsure.”*

1. Contraceptives cause disabilities/birth defects in children.
2. The string on the IUD hurts a man's penis.
3. An IUD can move around inside a woman and hurt her.
4. Contraceptives diminish sex drive.

Item response options: 3-point scale, where Agree = 3, Unsure = 2, Disagree = 1

The index is constructed by summing the item scores. The index score range is 4-12, and a higher score indicates less accurate knowledge about family planning.

**Family Planning Approval Index** (used with men and women)

*(Interviewer) “Now I would like to read you some statements about what you may think about those who use family planning methods, for each one can you tell me whether your agree, disagree or are unsure.”*

1. In general, I approve of couples using family planning to avoid or delay a pregnancy.
2. I think that most of my friends in this community would approve of couples using family planning to avoid or delay a pregnancy.
3. I think that my spouse would approve of couples using family planning to avoid or delay a pregnancy.
4. I approve of a young recently married couple with no children using family planning to avoid or delay a pregnancy.
5. I approve of a young unmarried woman having access to family planning.

Item response options: 5-point scale, where Approval = 1 and Disapproval = 2.

The index is constructed by summing the item scores. The index score range is 5‐15, and a higher score indicates a higher level of approval for family planning.
