# Supplementary material for: Community Dialogue to Shift Social Norms and Enable Family Planning: An Evaluation of the Family Planning Results Initiative in Kenya
Source: PLoS One. 2016 Apr 28;11(4):e0153907. doi: 10.1371/journal.pone.0153907 (PMC4849797; doi:10.1371/journal.pone.0153907)
Supplement: S5 File — (DOC) [file pone.0153907.s005.doc]

**CODEBOOK RI/CARE PROJECT – PLOS ONE**

| **Variable name** | **Coding** | **Variable definition** | **Collected for women** | | **Collected for men** | |
| --- | --- | --- | --- | --- | --- | --- |
|  |  |  | Baseline | Endline | Baseline | Endline |
| agef | <25…………….1  25-29………….2  30-34……...…..3  35+…………….4 | Respondent’s age (years) | x | x | x | x |
| agedif | <5………………1  5-9……………..2  10-14……….....3  15+………….…4 | Age difference between male and female partners (years) | x | x | x | x |
| religionf | Protestant……….1  Catholic………....2  Other…………….3  None/missing…...4 | Respondent’s religion | x | x | x | x |
| educ | *continuous* | Respondent’s education (single years) | x | x | x | x |
| speduc | *continuous* | Partner’s education (single years) | x | x | x | x |
| cash | Yes………..1  No…………0 | Respondent works outside home for cash | x | x | x | x |
| moneyspcash | No cash earned…0  All cash……….00..1  Some cash…..….2  Missing……….....3 | Woman makes decisions about the money she earns | x | x | x | x |
| maledominance | *continuous* | Gender beliefs scale score |  |  | x | x |
| wdm | *continuous* | Participation in household decision making scale score |  | x |  | x |
| isc | *continuous* | Interspousal communication scale score |  | x |  | x |
| intendpreg | Wants pregnancy in next 12 months...........1  Wants to delay pregnancy for ≥ 1 year.............................2  Wants no more children.......................3  Does not think about/not sure/not applicable...................4 | Pregnancy intentions | x | x | x | x |
| mythscore | *continuous* | Family planning beliefs score | x | x | x | x |
| FPnum | *continuous* | Number of family planning methods known | x | x | x | x |
| permfp | Agrees………..............1  Disagrees/unsure….…0 | Believes woman can use family planning without partner’s permission |  | x |  | x |
| suggcondom | Agrees………..............1  Disagrees/unsure….…0 | Believes woman can suggest use of condoms | x | x |  |  |
| approve | *continuous* | Family planning use approval score | x | x | x | x |
| sefp | *continuous* | Family planning self-efficacy scale score |  | x |  |  |
| knowFPplace | Yes………..1  No…………0 | Knows where to get a family planning method |  |  |  | x |
| RI_exposure_any | Yes………..1  No…………0 | Exposure to the intervention (any topic) |  | x |  | x |
| exp | *continuous* | Number of topics of exposure |  | x |  | x |
| fp_exp | Yes………..1  No…………0 | Exposure to family planning discussions during intervention |  | x |  | x |
| currentuse_any | Yes………..1  No…………0 | Current use of family planning (any method) | x | x | x | x |
| currentuse_modern | Yes………..1  No…………0 | Current use of modern family planning (modern method) | x | x | x | x |
| year | Baseline......2009  Endline.......2012 | Survey round | x | x | x | x |
